# Burnout among Brazilian Dentists during the COVID-19 Pandemic: A Cross-sectional Study

**DOI:** 10.2174/17450179-v19-e230717-2023-1

**Published:** 2023-08-02

**Authors:** Juliane Kely F. Silva, Fernando M. Carvalho, Carolina F. Azevedo, Marcos Vinicius S. Silva, Arthur Igor C. Lima, Liliane Lins-Kusterer

**Affiliations:** 1 Department of Social and Preventive Medicine, Universidade Federal da Bahia, State of Bahia, Brazil

**Keywords:** COVID-19, Dentists, Professional burnout, Anxiety, Quality of life, Occupational health

## Abstract

**Background:**

This article identifies the factors associated with burnout among dentists in the state of Bahia, Brazil, during the COVID-19 pandemic.

**Methods:**

A cross-sectional study, conducted between November 2020 and February 2021, of 251 dentists, selected by snowball sampling. Sociodemographic, occupational, and epidemiological information was collected in an electronic form. Burnout was evaluated through the Maslach Burnout Inventory; anxiety through the Beck Anxiety Inventory; and quality of life through the WHOQol-BREF questionnaire. Multiple linear regression was used to identify factors associated with variations in levels of the three burnout dimensions.

**Results:**

The mean levels of the burnout dimensions were: Emotional Exhaustion: 28.9 ± 8.9 (SD); Depersonalization = 8.2 ± 6.3; and Personal Accomplishment: 28.0 ± 6.2. The proportion of professionals with high Emotional Exhaustion (≥ 30 cut-off score), high Depersonalization (≥ 12 cut-off score), and low Personal Accomplishment (≤ 33 cut-off score) were 43.4%, 26.3% and 81.3%, respectively. Burnout components were associated with high anxiety; lower levels of the physical, psychological, social relations, and environmental dimensions of quality of life; less time working as a dentist; increased alcohol consumption during the pandemic, feeling safe when using personal protective equipment, and support from work fellows.

**Conclusion:**

The dentists investigated showed moderate levels of Emotional Exhaustion and Depersonalization, and very low levels of Personal Accomplishment. The prevention and mitigation of burnout syndrome among dentists during a pandemic should consider several factors, such as quality of life, amount of time in the profession, use of personal protective equipment, support from fellow dentists, and alcohol consumption.

## 1 INTRODUCTION

During the COVID-19 pandemic, dentists were heavily exposed to several stressors, such as the need to operate in work environments with a high risk of coronavirus infection and inadequate working conditions [1]. Dentists usually have intense contact with their patients, through exposure to saliva, blood, and other body fluids. The use of high-speed drills can produce aerosols that increase the risk of chemical and biological contamination [2].

Dentists’ exposure to occupational psychosocial stressors can lead to elevated levels of subjective overload and psychological distress associated with burnout syndrome [3, 4]. Burnout results from chronic workplace stress that has not been successfully managed. Burnout, coded as “QD85” in the 11th Revision of the International Classification of Diseases, is considered an occupational phenomenon and is not classified as a medical condition [5].

The Maslach Burnout Inventory (MBI) was developed as a research instrument to assess burnout on a continuum, ranging from low to high and encompassing three dimensions: Emotional Exhaustion, Depersonalization, and reduced Personal Accomplishment. The research perspective of a continuum differs from that of a practitioner, who might view burnout as a discrete state, like a medical disease. This has led people to seek a measure that will provide a dichotomous “diagnosis” of burnout. The scientific literature about the MBI also uses arbitrary statistical “cut-off” scores to identify people demonstrating “high” burnout, in the way one might refer to the ‘top third’ of a large population. Although useful for assessing the overall pattern of a group, such “cut-off” scores do not have any diagnostic validity [6].

There is scant literature about burnout among dentists during the COVID-19 pandemic, strictly measured according to the levels of the three MBI dimensions, as recommended by its developers.^6^ A comprehensive search performed by the authors found only two studies. One study of 442 dentists from Turkey found mean (± standard deviation) scores of EE= 20.83 ± 8.46; DP = 7.58 ± 4.15; and PA = 9.41 ± 4.65. Bivariate analysis revealed that EE was moderately and positively correlated with general, personal and occupational stress levels and with a working knowledge of the COVID-19 pandemic; DP was associated with male gender, smoking, using public transport, and working in private clinics; PA was associated with using public transport [7]. Another study of 218 dentists engaged in primary dental care in Scotland reported mean scores of EE = 31.70 ± 16.31; and DP = 9.00 ± 5.24. Unfortunately, this article did not provide information about factors associated with burnout [8]. Prior to the pandemic, many studies identified these factors as: younger age, male gender, high job strain, high working hours [9, 10]. A study of dentists from Northern Ireland reported that time pressure, financial worries, and dealing with difficult patients correlated positively with both EE and DP: 0.30 > r < 0.62.10.

Brazil ranks second in the number of deaths (687,907 deaths) due to COVID-19 [11], despite representing only 2.7% of the global population [12]. Therefore, it is of utmost importance to investigate how the COVID-19 pandemic affected the mental health of dentists to provide planning and strategies to improve their working conditions. Gaps in the scientific literature about burnout among dentists and their working conditions during the pandemic reveal the relevance of this study.

The study aimed to identify factors associated with burnout among dentists in the state of Bahia, Brazil, during the COVID-19 pandemic.

## 2 MATERIALS AND METHODS

### 2.1 Study Design, Setting, and Participants

Between November 2020 and February 2021 of the COVID-19 pandemic, a cross-sectional study was carried out with dentists from the state of Bahia, Brazil. All active dentists who accepted the invitation to participate in the study and signed an informed consent form were included. Convenience virtual snowball sampling was employed. A link to a Google Form questionnaire was made available *via* WhatsApp, email, and Facebook dentist groups. Dentists who responded to the questionnaire were asked to forward the link to other colleagues in order to maximize participation.

Information was collected about a) sociodemographic aspects: sex, age, marital status, skin colour, monthly family income (1 USD = 5.187 Brazilian Reals), amount of time worked as a dentist, and employer economic sector (public, private or both); b) aspects related to COVID-19 and their occupation: diagnosed with COVID-19, afraid of catching COVID-19, avoids seeing patients suspected of having COVID-19, increased alcohol consumption during the pandemic, much higher work demands due to COVID-19, accomplishes all work tasks, feels safe when using personal protective equipment at work, has support from fellow dentists, has support from other health professionals, feels qualified to provide care to COVID-19 cases, and has put their dentistry practice on hold because of the pandemic; c) Quality of life, evaluated by the WHOQoL-BREF questionnaire. This 26-question instrument has four domains: physical, psychological, social relations, and environmental, with scores varying from 4 to 20, where higher scores represent better quality of life [13]. The WHOQoL-BREF has been translated and validated in Brazil [14]. In a sample of 3,574 university staff from a Brazilian university, the questionnaire showed acceptable levels of internal consistency and test-retest reliability [15]; d) Anxiety, measured by the Beck Anxiety Inventory (BAI). The 21 items of this questionnaire are classified into a 4-point Likert scale, with options varying from 0 to 3. Total scores can vary from 0 to 63, with higher total scores indicating more severe anxiety symptoms [16]. This questionnaire has been translated into Brazilian Portuguese and validated, presenting good psychometric properties [17, 18]; and e) Burnout, measured by the Maslach Burnout Inventory (MBI), along three dimensions: Emotional Exhaustion, Depersonalization, and Personal Accomplishment. The 22 self-reported items are classified in Likert-type scales that can vary from 0 to 6. The maximum scores for Emotional Exhaustion, Depersonalization, and Personal Accomplishment are 54, 30, and 40, respectively. Dimension scoring was performed in line with the MBI developers [6].

### 2.2 Data Analysis

Dependent variable: Burnout; the scores for each of the three MBI dimensions - Emotional Exhaustion (EE), Depersonalization (DP), and Personal Accomplishment (PA) - were taken as continuous variables. The proportion of dentists with high EE: (score ≥ 30); high DP: (score ≥ 12); and low PA (score ≤ 33) was classified according to published normative cut-off levels [19].

Independent variables: The questions about COVID-19 were originally collected from a Likert-type scale with five options that were subsequently codified as: Frequently and Sometimes = Yes; Neutral, Rarely, and Never = No.

The correlation between age and monthly family income and MBI domain scores was evaluated by calculating the Pearson linear correlation coefficient. Differences between the means of an MBI domain according to each independent variable were measured by independent t-tests. Variables that reached a P level of < 0.20 were selected to compose three multiple linear regression models, where Emotional Exhaustion, Depersonalization, and Personal Accomplishment were the respective dependent variables, using the backward method. Only those variables that reached a P level of < 0.05 remained in the final adjusted models.

In the studentized residuals analysis, cases presenting ± 3.000 standard deviations were classified as outliers and excluded from the model. Collinearity between independent variables was evaluated through tolerance statistics. Values close to zero denote strong collinearity and indicate that the independent variable has a linear performance similar to another variable or variables in the model.

This study used convenience, non-probabilistic sampling, which prevents the use of statistical inference based on P-values or confidence intervals. P-values obtained from t-tests were simply used for selecting variables for the multiple linear regression. The multiple linear regression technique was used to adjust the raw and standardized regression coefficients (also known as the BETA coefficients) obtained for the predictive variables in each model without making statistical inferences. BETA coefficients enable direct comparisons between the variables in the model since they are measured in Z-score scales.

The reliability of the MBI, WHOQoL-BREF, and BAI questionnaires was evaluated through the internal consistency of the domains using Cronbach’s alpha: 0 to 1, where 0 to 0.21 indicates little consistency; 0.21 to 0.40, average; 0.41 to 0.60, moderate; 0.61 to 0.80, substantial; and 0.81 to 1.0, nearly perfect consistency [20].

Statistical data processing was performed using the Statistical Package for the Social Sciences™ - SPSS, version 25 (IBM Corp, Armonk, NY, USA).

## 3 RESULTS

The 251 dentists we investigated had a mean age of 32.1 years (SD = 8.3) and a monthly family income of 1,081 USD (SD = 939). The mean scores and Cronbach’s alpha values for the burnout domain, the quality of life domain, and anxiety are presented in Table **1**.

The study population was predominantly female (72.3%), lived with a partner (68.5%), had graduated as a dentist less than 6 years previously (57.0%), worked in the private sector (78.7%), had not caught COVID-19 (94.4%), but was afraid of doing so (73.7%), and avoided seeing patients suspected of having the disease (70.5%). During the pandemic, dentists referred to increased consumption of alcohol (66.9%), and much higher work demands (86.5%), although they continued to accomplish all their work tasks (77.7%), felt safe using the personal protective equipment at work (61.8%), were supported by fellow dentists (66.9%), but not by other health professionals (68.1%), experienced reduced quality in their social relationships (89.6%), and put their dentistry on hold because of the pandemic (76.5%) (Table **2**).

The bivariate analysis of the dependent variables Emotional Exhaustion, Depersonalization, and Personal Accomplishment, according to the categorical and continuous variables, are presented in Tables **2** and **3**, respectively.

A multiple linear regression equation showed that the variation in mean Emotional Exhaustion was strongly and positively associated with anxiety. An estimated increase of 4.000 points in the Emotional Exhaustion score was associated with an increase of one unit in the anxiety score, controlling for the effects of the other model variables. Furthermore, an estimated decrease of 0.827 unit in the Emotional Exhaustion score was associated with each increase in the WHOQoL-BREF Physical score. The WHOQoL-BREF Social relations score, as well as the score for feeling safe when using personal protective equipment at work, were also strongly and negatively (inversely) associated with Emotional Exhaustion. The BETA coefficients enable direct comparisons between the different independent variables included in the model. Therefore, the independent variable associated with the greatest variation in Emotional Exhaustion was WHOQoL-BREF Physical (-0.245), followed by the WHOQoL-BREF Social relations (-0.231), anxiety (0.204), and feeling safe when using personal protective equipment at work (-0.155), in decreasing order of strength (Table **4**).

Depersonalization was inversely associated with the WHOQoL-BREF Social relations and Environmental variables, amount of time working as a dentist, increased alcohol consumption during the pandemic, and feeling safe when using personal protective equipment at work, and positively associated with anxiety. The BETA coefficient revealed that the WHOQoL-BREF Social relations variable had the most influence on the linear variation in the Depersonalization scores (Table **4**).

The Personal Accomplishment mean score was positively and strongly associated with WHOQoL-BREF Psychological score (BETA = 0.292), and having support from fellow dentists (BETA = 0.209); and inversely associated with anxiety (BETA = -0.131).

The studentized residues identified two outliers in the multivariate equation that had Depersonalization as its dependent variable and one outlier in the equation for Personal Accomplishment; these were excluded from the analyses. The multivariate models were well adjusted to the data for the three burnout domains, as revealed by an ANOVA P-value of <0.001. The adjusted R^2^ coefficients show that the independent variables included in the models explain 33.5%, 23,4%, and 19.4% of the variation in the mean levels for Emotional Exhaustion, Depersonalization, and Personal Accomplishment, respectively. The Durbin-Watson statistic values for the three models fell within the acceptable range (1.5 to 2.5). Finally, the Tolerance statistics were high for the three models, ranging from 0.553 to 0.973, indicating negligible collinearity (Table **4**).

Among these 251 dentists, the proportions of professionals with high Emotional Exhaustion (≥ 30), high Depersonalization (≥ 12), and low Personal Accomplishment (≤ 33) were 43.4% (n = 109), 26.3% (n = 66), and 81.3% (n = 204), respectively.

## 4 DISCUSSION

### 4.1 Levels of Burnout among Dentists during the COVID-19 Pandemic

All three burnout domains - Emotional Exhaustion, Depersonalization, and Personal Accomplishment, presented satisfactory internal consistency, as revealed by substantial or nearly perfect Cronbach’s alpha values.

In this study, Brazilian dentists had higher means for EE, DP, and PA than dentists from Turkey [7] and lower means for EE and DP than those from Scotland [8].

Some of the studies about burnout among dentists during the COVID-19 pandemic only applied certain items from the complete set recommended by the MBI manual [6] in order to define EE [21] and PA [22]. Unfortunately, the procedures used in these studies preclude comparison with our findings.

Studies of other health professionals used comparable methodologies to measure burnout during the COVID-19 pandemic in the state of Bahia, Brazil. One study evaluated 223 physicians working on the COVID-19 frontline and reported mean levels of EE = 27.5 ± 11.6; DP = 6.4 ± 5.5; low PA = 35.6 ± 7.9 [23]. Another study evaluated 752 multi-professional residents during the early days of the pandemic and reported mean levels of EE = 29.6 ± 11.8; DP = 7.0 ± 5.6; low PA = 30.9 ± 7.3 [24]. The mean levels of burnout reported in these two studies were similar to those found in the dentists in this study: EE = 28.9 ± 8.9; DP = 8.2 ± 6.3; and PA = 28.0 ± 6.2.

### 4.2 Factors associated with Burnout among Dentists during the COVID-19 Pandemic

Anxiety was the only variable in this study that was strongly associated with all three burnout dimensions. Following this, the association between burnout and the other variables investigated, which are strictly related to chronic workplace stress, need to be discussed.

In this study, the mean scores for several quality of life domains were strongly associated with the mean scores of all three burnout dimensions. Such findings were expected, given the broad and complex facets encompassed by the quality of life domains which, in turn, are intrinsically linked to the chronic workplace stress that defines burnout. A study of 185 physicians without anxiety, working on the COVID-19 frontline in the state of Bahia, Brazil, presented a moderate to high degree of correlation between the four WHOQol domains and EE (-0.37 < r < 0-0.56), DP (-0.28 < r <0.41), and PA (0.30 < r < 0.51) [23]. Another study of 442 Brazilian multi-professional residents without anxiety, working during the COVID-19 pandemic, reported moderate correlations between EE and WHOQol Physical (r = -0.37), and between EE and WHOQoL Psychological (r = -0.33) [24]. In both of these studies, “without anxiety” was defined by a score <16 according to the Beck Anxiety Inventory [25].

Feeling safe when using personal protective equipment at work was associated with lower Emotional Exhaustion (-0.149 points) and Depersonalization (-0.116 points) scores. The sensation of safety at work is essential for coping with the fear of contracting the virus and transmitting it to patients and loved ones, decreasing stress and consequent burnout, particularly among professionals on the frontline of a new and unpredictable pandemic. Feeling safe at work when using personal protective equipment was referred to by 62.3% of dentists, [26] 78.5% of physicians [24], and 79.5% of nursing professionals [27] working on the frontline against the COVID-19 pandemic in Brazil.

Dentists with six or more years of professional practice presented less Depersonalization. Age can be taken as a proxy for time in the profession, corroborating the finding that being young is an important factor associated with burnout [9].

Increased alcohol consumption by dentists during the pandemic was associated with a higher mean level of Depersonalization. The Emotional Exhaustion and Depersonalization burnout dimensions were negatively related to the regular use of alcohol among Lithuanian dentists [28]. In Brazil, increased alcohol consumption during the pandemic was mentioned by 36.5% of dentists [26], 37.2% of physicians [23], 47.8% of nursing professionals [27], and 51.1% of multi-professional residents [24]. Increased alcohol consumption could represent both adaptive and maladaptive methods for coping with the stress of the pandemic [4]. Alcohol consumption merits special attention because it is a potentially modifiable factor in the prevention of burnout among dentists during this pandemic.

In this study, dentists who referred to support from their fellow dentists scored higher for Personal Accomplishment. Personal Accomplishment was also inversely associated with anxiety and positively associated with the psychological dimension of quality of life. Furthermore, these findings must be interpreted in a context in which 81.3% of dentists can be classified as demonstrating low Personal Accomplishment, according to the cut-off level [19].

### 4.3 Proportion of Dentists with High Levels of Burnout

A recent study of dentists engaged in primary dental care in Scotland reported that the proportions of dentists with high “recognized published cut-off values” for EE and DP were 55.6% and 32.2%, respectively. Unfortunately, the study did not specify these cut-off values [8]. A study of 260 Canadian dental hygienists as they returned to work following service interruption due to the COVID-19 pandemic found EE (≥ 27) = 65%, DP (≥ 10) = 34%, and PA (≤ 33) = 24% [29]. Another study of dental hygienists from California carried out prior to the pandemic, reported EE (>27) = 30.0%, DP (>10) = 11.3%, and PA (<33) = 41.1% [30]. Our study of Brazilian dentists reported the following proportions of high EE (≥ 30) = 43.4%; high DP (≥ 12) = 26.3%; and low PA (≤ 33) = 81.3%. Other studies have reported different or non-specified cut-off levels, precluding valid comparisons with our findings. Despite this limitation, the very high proportion of Brazilian dentists with low PA remains noteworthy. Summing up the evidence, this study concludes that the dentists we investigated showed moderate levels of Emotional Exhaustion and Depersonalization but very low levels of Personal Accomplishment. The prevention and mitigation of burnout syndrome among dentists during a pandemic must take into account of several factors, such as quality of life, time spent in the profession, use of personal protective equipment, support from fellow dentists, and alcohol consumption.

This study has certain important limitations: a small number of individuals was investigated; selection bias could derive from the convenience virtual snowball sampling used; in the context of a cross-sectional study such as this, it is impossible to determine the directional relationship between risk factors and burnout. Finally, other factors related to the prevalence of burnout, stress disorders, and depression were not investigated in our model.

## CONCLUSION

In conclusion, this study found that burnout components were associated with high anxiety; poorer levels of the physical, psychological, social relations, and environmental dimensions of quality of life; less time spent working as a dentist; alcohol consumption, use of personal protective equipment, and support from work fellows. The prevention and mitigation of burnout syndrome among dentists during a pandemic must consider these personal, social, and occupational aspects.

## AUTHORS’ CONTRIBUTIONS

J.K.F.S., C.F.A., M.V.S.S., A.I.C.L, and L.L-K conceived the ideas; J.K.F.S., C.F.A., M.V.S.S., and A.I.C.L. collected the data; F.M.C., L.L-K and J.K.F.S. analysed the data; and J.K.F.S. and F.M.C. led the writing. All authors revised and approved the final version and agreed to be accountable for all aspects of the work.

## Figures and Tables

**Table 1:** Mean, standard deviation, minimum and maximum values of scores of burnout, quality of life, anxiety, age, monthly family income, and reliability (Cronbach’s alpha) among 251 dentists from State of Bahia, Brazil, November, 2020 - February, 2021.

**Variable**	**Mean**	**Sd.**	**Min.**	**Max.**	**Cronbach’s Alpha**
Burnout (Maslach Burnout Inventory)	-	-	-	-	-
Emotional exhaustion	28.9	8.9	6.0	51.0	0.83
Depersonalization	8.2	6.3	0.0	30.0	0.94
Personal accomplishment	28.0	6.2	8.0	40.0	0.93
Quality of life (WHOQoL-BREF domain)	-	-	-	-	-
Physical	14.6	2.6	5.6	20.0	0.79
Psychological	14.1	2.8	6.0	19.3	0.81
Social relations	13.7	3.3	4.0	20.0	0.71
Environmental	13.8	2.5	7.5	19.0	0.77
Anxiety (Beck Anxiety Inventory)	12.1	10.0	0.0	45.0	0.93
Age (Years)	32.1	8.3	22	65	-
Monthly family income (USD)	1,081	939	116	7,712	-

**Table 2:** Burnout domains scores (mean and standard deviation) according to independent variables of 251 dentists, State of Bahia, Brazil, November, 2020 - February 2021.

**Variable**	**N**	**%**	**Emotional Exhaustion**			**Depersonalization**			**Personal Accomplishment**		
			**Mean**	**Sd.**	**P**	**Mean**	**Sd.**	**P**	**Mean**	**Sd.**	**P**
Sex	-	-	-	-	0.934	-	-	0.264	-	-	0.141
Feminine	184	73.3	28.9	9.1	-	7.9	6.1	-	28.4	6.3	-
Masculine	67	26.7	28.8	8.4	-	8.9	6.7	-	27.1	5.8	-
Marital status	-	-	-	-	0.284	-	-	0.352	-	-	0.812
Without partner	79	31.5	29.8	8.7	-	8.7	6.1	-	27.9	6.1	-
With partner	172	68.5	28.5	90	-	7.9	6.4	-	28.1	6.2	-
Skin color	-	-	-	-	0.859	-	-	0.531	-	-	0.900
Black/Brown	137	54.6	28.7	8.9	-	8.4	6.0	-	28.1	5.9	-
White/Yellow	114	45.4	29.1	9.0	-	7.9	6.6	-	28.0	6.5	-
Time as a dentist (years)	-	-	-	-	0.112	-	-	0.004	-	-	0.059
0 - 5	143	57.0	29.7	8.8	-	9.1	6.2	-	27.8	6.2	-
6 or more	108	43.0	27.9	8.9	-	6.8	6.2	-	29.3	6.2	-
Employer economy sector	-	-	-	-	0.376	-	-	0.857	-	-	0.491
Private	124	49.4	28.3	8.2	-	8.2	6.2	-	28.0	5.7	-
Public	56	22.3	30.3	9.4	-	8.4	6.3	-	27.4	6.5	-
Public and private	71	28.3	28.8	9.6	-	7.8	6.6	-	28.7	6.6	-
Diagnosed with COVID-19	-	-	-	-	0.416	-	-	0.773	-	-	0.143
No	237	94.4	28.8	8.9	-	8.1	6.2	-	27.9	6.2	-
Yes	14	5.6	30.8	9.2	-	8.6	7.6	-	30.4	5.8	-
Afraid of catching COVID-19	-	-	-	-	0.212	-	-	0.060	-	-	0.264
No	66	26.3	27.7	8.4	-	6.9	6.5	-	28.8	6.0	-
Yes	185	73.7	29.3	9.1	-	8.6	6.2	-	27.8	6.3	-
Avoid attending patients suspected of COVID-19	-	-	-	-	0.087	-	-	0.067	-	-	0.285
No	74	29.5	30.4	7.9	-	9.3	6.4	-	27.3	7.1	-
Yes	177	70.5	28.3	9.2	-	7.7	6.2	-	28.3	5.7	-
Increased alcohol consumption during the pandemic	-	-	-	-	0.802	-	-	0.097	-	-	0.118
No	168	66.9	28.8	9.3	-	7.7	6.2	-	28.5	6.4	-
Yes	83	33.1	29.1	8.1	-	9.1	6.4	-	27.2	5.7	-
Work demands much more from me due to COVID-19	-	-	-	-	0.089	-	-	0.864	-	-	0.295
No	34	13.5	26.5	8.1	-	8.0	7.2	-	27.0	6.8	-
Yes	217	86.5	29.3	99.0	-	8.2	6.2	-	28.2	6.1	-
Accomplishes all work tasks	-	-	-	-	0.159	-	-	0.028	-	-	0.025
No	56	22.3	30.4	9.1	-	9.8	7.7	-	26.4	6.0	-
Yes	195	77.7	28.5	8.8	-	7.7	6.0	-	28.5	6.2	-
Feels safe with personal protective equipment at work	-	-	-	-	<0.001	-	-	0.001	-	-	0.012
No	96	38.2	32.0	9.3	-	9.9	6.9	-	26.8	5.8	-
Yes	155	61.8	27.0	8.1	-	7.1	5.7	-	28.8	6.3	-
Has support of fellow dentists	-	-	-	-	0.112	-	-	<0.001	-	-	<0.001
No	83	33.1	30.2	8.6	-	10.4	6.8	-	25.8	6.3	-
Yes	168	66.9	28.3	9.0	-	7.1	5.8	-	29.1	5.8	-
Has support of other health professionals	-	-	-	-	0.038	-	-	0.004	-	-	0.016
No	171	68.1	29.7	8.9	-	9.1	6.5	-	27.4	6.4	-
Yes	80	31.9	27.2	8.7	-	6.3	5.3	-	29.4	5.4	-
Feels qualified to provide care to COVID-19 cases	-	-	-	-	0.426	-	-	0.259	-	-	0.015
No	129	51.4	29.3	9.4	-	8.6	6.2	-	27.1	6.3	-
Yes	122	48.6	28.4	8.4	-	7.7	6.4	-	29.0	5.9	-
Reduced the quality of social relationships	-	-	-	-	0.144	-	-	0.092	-	-	0.583
No	26	10.4	26.5	7.8	-	6.2	5.1	-	28.7	6.5	-
Yes	225	89.6	29.2	9.0	-	8.4	6.4	-	28.0	6.1	-
Put dentistry practice on hold because of the pandemic	-	-	-	-	0.326	-	-	0.749	-	-	0.390
No	59	23.5	27.9	8.1	-	8.4	5.5	-	27.4	7.0	-
Yes	192	76.5	29.2	9.1	-	8.1	6.5	-	28.2	5.9	-

**Table 3:** Pearson correlation coefficient (R) between burnout domains and independent variables in 251 dentists from the State of Bahia, Brazil, November, 2020 - February, 2021.

**Variable**	**Emotional Exhaustion**		**Depersonalization**		**Personal Accomplishment**	
	**R**	**P-value**	**R**	**P-value**	**R**	**P value**
Age, years	-0.098	0.123	-0.124	0.050	0.057	0.369
Monthly family income, USD	-0.132	0.037	-0.065	0.302	0.017	0.783
Anxiety, Beck anxiety inventory	0.502	<0.001	0.393	<0.001	-0.295	<0.001
Quality of life, WHOQoL-BREF	-	-	-	-	-	-
Physical	-0.505	<0.001	-0.320	<0.001	0.271	<0.001
Psychological	-0.507	<0.001	-0.382	<0.001	0.386	<0.001
Social relations	-0.421	<0.001	-0.347	<0.001	0.238	<0.001
Environmental	-0.378	<0.001	-0.328	<0.001	0.190	<0.003

**Table 4:** Regression coefficients of multiple linear regression models having the scores of the three burnout domains of the Maslach Burnout Inventory as the dependent variable according to independent variables of dentists from the State of Bahia, Brazil. November, 2020 - February, 2021.

**Independent Variable (Referent)**	**Emotional Exhaustion (n=251)**				**Depersonalization (n=249)**				**Personal Accomplishment (n=250)**			
	**b^a^**	**b_(EP)_^b^**	**Beta^c^**	**p**	**b**	**b_(EP)_**	**Beta**	**p**	**b**	**b_(EP)_**	**Beta**	**p**
Anxiety. Beck anxiety inventory	4.000	1.174	0.204	0.001	2.076	0.776	0.156	0.008	-1.739	0.838	-0.131	0.039
WHOQoL-BREF - Physical	-0.827	0.234	-0.245	<0.001	-	-	-	-	-	-	-	-
WHOQoL-BREF - Psychological	-	-	-	-	-	-	-	-	0.637	0.138	0.292	<0.001
WHOQoL-BREF - Social relations	-0.627	0.161	-0.231	<0.001	-0.437	0.113	-0.243	<0.001	-	-	-	-
WHOQoL-BREF - Environmental	-	-	-	-	-0.403	0.160	-0.164	0.013	-	-	-	-
Time as a dentist (0 - 5 years)	-	-	-	-	-1.394	0.694	-0.115	0.046	-	-	-	-
Increased alcohol consumption during the pandemic (No)	-	-	-	-	1.749	0.714	0.138	0.015	-	-	-	-
Feels safe with personal protective equipment at work (No)	-2.831	0.970	-0.155	0.004	-0.143	0.707	-0.116	0.044	-	-	-	-
Has support of fellow dentists (No)	-	-	-	-	-	-	-	-	2.674	0.738	0.209	<0.001
Intercept	50.125	3.189	-	<0.001	19.843	2.178	-	<0.001	17.856	2.091	-	<0.001
Durbin-Watson statistics	1.867				1.953				1.862			
Adjusted R^2^	0.335				0.234				0.194			
ANOVA	<0.001				<0.001				<0.001			
Tolerance (1-R^2^)	0.553-0.943				0.726-0.979				0.801-0.973			

**Note:** ^a^ - Non standardized regression coefficient; ^b^ - Standard error of b; ^c^ - Standardized regression coefficient

## Data Availability

The data supporting the findings of the article is available in the figshare Repository at figshare.com, reference number doi 10.6084/m9.figshare.23528397.

## LIST OF ABBREVIATIONS

EE: = Emotional Exhaustion
DP: = Depersonalization
PA: = Personal Accomplishment
ANOVA: = Analysis of Variance
COVID-19: = Coronavirus Disease 2019
WHOQoL: = World Health Organization Quality of Life
